# Strengthening supply chains for pathogen genomic surveillance in Asia

**DOI:** 10.1136/bmjgh-2025-019241

**Published:** 2026-02-06

**Authors:** Anne-Claire Stona, Yoong Khean Khoo, La Moe, Suci Wulandari, Shreya Agoramurthy, Marya Getchell, Tze-Minn Mak, Junxiong Pang, Elyssa Jiawen Liu, Shurendar Selva Kumar, John CW Lim, Gavin J D Smith, Alexandra Bertholet, Arika Garg, Steven Harsono, Maeve Magner, Firdausi Qadri, Tahmina Shirin, Lucia Rizka Andalucia, Syarifah Liza Munira, Phonepadith Xangsayarath, Matthew T Robinson, Swe Setk, Hlaing Myat Tu, Govindakarnavar Arunkumar, Runa Jha, Afreenish Amir, Aamer Ikram, Imran Nisar, Timothy Dizon, Cynthia Saloma, Neelika Gathsaurie Malavige, Ruklanthi De Alwis, Paul M Pronyk

**Affiliations:** 1 SingHealth Duke-NUS Global Health Institute, Duke-NUS Graduate Medical School, Singapore; 2 Centre for Outbreak Preparedness, Duke-NUS Graduate Medical School, Singapore; 3 Centre of Regulatory Excellence, Duke-NUS Graduate Medical School, Singapore; 4 Centre for Evidence and Implementation, Singapore; 5 Agency for Science Technology and Research, Singapore; 6 Emerging Infectious Diseases Programme, Duke-NUS Graduate Medical School, Singapore; 7 Independent Consultant, Washington, DC, USA; 8 Clinton Health Access Initiative, Boston, Massachusetts, USA; 9 Independent Consultant, Singapore; 10 International Centre for Diarrhoeal Disease Research Bangladesh, Dhaka, Bangladesh; 11 Institute of Epidemiology Disease Control and Research Bangladesh, Dhaka, Bangladesh; 12 Ministry of Health of the Republic of Indonesia, Jakarta, Indonesia; 13 Department of Communicable Disease Control, National Center for Laboratory and Epidemiology (NCLE), Ministry of Health Lao PDR, Vientiane, Lao People’s Democratic Republic; 14 Lao-Oxford-Mahosot Hospital-Wellcome Trust Research Unit, Microbiology Laboratory, Mahosot Hospital, Vientiane, Lao People’s Democratic Republic; 15 National Health Laboratory, Department of Medical Service, Ministry of Health Myanmar, Yangon, Myanmar; 16 Department of Medical Research, Ministry of Health Myanmar, Naypyitaw, Myanmar; 17 World Health Organisation Nepal, Kathmandu, Nepal; 18 National Public Health Laboratory, Ministry of Health & Population, Teku, Kathmandu, Nepal; 19 National Institute of Health, Islamabad, Pakistan; 20 Aga Khan University, Karachi, Pakistan; 21 Research Institute for Tropical Medicine, Muntinlupa, Philippines; 22 University of the Philippines Manila, Manila, Philippines; 23 Department of Immunology and Molecular Medicine, Faculty of Medical Sciences, University of Sri Jayewardenepura, Nugegoda, Sri Lanka

**Keywords:** Public Health, Global Health, Health policy, Health systems, Health policies and all other topics

## Abstract

**Introduction:**

While pathogen genomics using next-generation sequencing (NGS) has been recommended by the WHO as an essential tool for national communicable disease surveillance programmes, procurement and supply chain management (PSM) systems for this new technology are still evolving. To assess the status of PSM systems for pathogen genomics, we examined perspectives from end-users and manufacturers across South and Southeast Asia.

**Methods:**

Between 2022 and 2023, a cross-sectional survey was conducted among institutional partners supporting pathogen genomics among primarily low- and middle-income countries in South and Southeast Asia. This was complemented by qualitative interviews with the major regional NGS manufacturers. A PSM framework was employed to assess sales, procurement, production, distribution and post-sales support. Analyses are expressed as proportions and means or medians for continuous variables.

**Results:**

A total of 42 partners across 13 countries, 3 genomics manufacturers and 22 laboratory personnel contributed data to this assessment. PSM challenges were reported by all countries and for all sequencing platforms. High costs of equipment and consumables were identified by 85% of respondents. Long equipment purchasing lead times and reagent re-supply times were reported by 69% and 77% of countries, respectively, with reagent resupply times averaging 8 weeks (IQR 6.2–9.0). Additional barriers included customs clearance, variability of import procedures, taxes and duties. Manufacturers reported a range of strategies to respond to PSM bottlenecks, including establishing regional hubs, distributor networks and financing schemes.

**Conclusion:**

Coordinated national and regional efforts are required to improve PSM systems for pathogen genomic sequencing to enhance timely early disease detection and response capacity in South and Southeast Asia.

**WHAT IS ALREADY KNOWN ON THIS TOPIC**

- Major global disparities exist in applying genomic sequencing to detect and respond to communicable disease threats.
- Inequalities exist in access and pricing, where countries with limited resources and greater outbreak risk pay 10-fold more than higher-income settings.

**WHAT THIS STUDY ADDS**

- Using a comprehensive multi-country mixed methods approach among both consumers and manufacturers, we identified key challenges, opportunities and actionable strategies to improve access to pathogen genomic sequencing technology.

**HOW THIS STUDY MIGHT AFFECT RESEARCH, PRACTICE OR POLICY**

- Our findings underscore the importance of coordinated national and regional efforts including ensuring transparent and affordable pricing for genomic commodities from suppliers, developing new forecasting tools to enhance cost efficiency, exploring innovative procurement models, implementing digital technology to monitor production of genomic sequencing equipment and streamlining and accelerating procurement processes (regulatory requirements, customs clearance, tax waivers) to improve access to pathogen genomic sequencing to enhance timely early disease detection and response capacity in South and Southeast Asia.

## Introduction

 The COVID-19 pandemic revealed the fragility of global health supply chains with major disruptions to essential health commodities. Supply chain disruptions are defined as ‘unplanned and unanticipated events that disrupt the normal flow of goods and materials within a supply chain’^1^ and health commodities such as personal protective equipment, medicine and vaccines were in short supply during the pandemic.^2^ These supply chain disruptions also affected other health commodities such as next-generation sequencing (NGS) equipment and consumables, which played an important role in informing public health interventions in the pandemic through tracking of the SARS-CoV-2 variants and transmission modelling.^3^ The WHO has developed recommendations for integrating NGS into national infectious disease surveillance programmes, especially for pathogens with pandemic and epidemic potential.^4^ Robust and efficient procurement and supply chain management (PSM) of genomics equipment and consumables is a prerequisite for such integration. This requires efficient coordination among raw material suppliers, platform manufacturers, third-party reagent manufacturers, distributors, consumer laboratories and governments to facilitate the generation of timely pathogen sequencing data to inform public health action.^5^

Wide global disparities exist in pathogen genomic surveillance capacity, with high-income countries contributing more sequences to public databases compared with lower-resourced settings.^6^ This is a result of gaps in infrastructure, staff capacity and resources. However, this could be under-reported as some countries with national capacities prefer not to share on publicly accessible datasets. Turnaround time from sample collection to submission time on international genomic databases is shorter in high-income countries compared with lower-resourced countries, highlighting inequalities in surveillance and laboratory system capacities.^7^ PSM challenges have also been put forth as a major barrier and potential driver of costs, limiting the potential for pathogen genomics to inform timely public health measures.^8 9^ Despite the cost of sequencing technologies decreasing over the past decade,^10^ substantial price inequities still exist. A study conducted by FIND in 2021 showed lower-resourced countries paying significantly higher costs per pathogen sequence.^11^ However, since the pandemic, there are ongoing market shaping efforts by FIND and the Global Fund to improve accessibility and affordability of genomic sequencing-related equipment. Import taxes and local distributor pricing have been previously identified as additional cost drivers in some settings.^12^ Furthermore, during the COVID-19 pandemic, global shortages faced by NGS manufacturers resulted in delayed delivery of pathogen sequencing-related equipment and reagents.^13^

Asia Pathogen Genomics Initiative is a coordination and capacity development platform that aims to accelerate the application of pathogen genomics across the region. We examined the status and challenges related to PSM and how this affects the end-user cost for pathogen genomics in South and Southeast Asia. Outcomes of this research are intended to inform national and regional strategies and policies to address PSM bottlenecks and cost barriers to support timely and equitable market access to sequencing equipment and reagents to advance early detection of novel and endemic pathogens.

## Methods

A cross-sectional quantitative survey was conducted among country partners contributing to pathogen genomic surveillance in South and Southeast Asia.^14^ This was combined with semi-structured qualitative interviews with Asia’s major NGS manufacturers, who are the primary providers of NGS-related equipment and supplies for pathogen genomics.

### Cross-sectional survey of pathogen sequencing product consumers

Detailed methodology for the multi-country cross-sectional survey of country partners (ie, consumers of NGS products) was previously described.^14^ Briefly, respondents were identified through national stakeholder consultations with public health and government institutes, selected for their expertise and experience in pathogen genomic sequencing. A questionnaire was generated based on an extensive synthesis of existing tools to assess current capacity and challenges related to pathogen genomics. It was administered to partners contributing to pathogen genomic surveillance in Bangladesh, Brunei Darussalam, Cambodia, Indonesia, Lao PDR, Malaysia, Myanmar, Nepal, Pakistan, Philippines, Sri Lanka, Thailand and Vietnam.^14^ A full list of participating national public health institutions, academic/research organisations, private laboratories and non-governmental organisations (NGOs) who contribute to pathogen genomics (ie, consumers) is included in online supplemental appendix 1. The questionnaire included a detailed assessment of PSM processes and challenges. Specifically, 19 indicators related to the sales, procurement, production, distribution and post-sales components of the supply chain were generated, with indicator definitions provided in table 1. Most questions were tailored to a specific manufacturing platform within each institution or country. A facilitated self-assessment process was used to administer the tool, with post-survey follow-up verification conducted to review findings and seek further clarity as needed.

**Table 1:** Summary of key supply chain and management barriers for pathogen genomic sequencing among resource-constrained consumer countries in the Asian region

Thematic area	Indicator	Definition	Value
Sales and operation planning	Sequencing platforms	Countries using respective platforms for pathogen genomic surveillance	Illumina=11 (85%) ONT=11 (85%) ThermoFisher=5 (38%) MGI/BGI=2 (15%)
	Cost of equipment	Countries reporting the cost of laboratory equipment (sequencing machines, sample storage equipment) as high-cost drivers	11 (85%)
	Cost of laboratory supplies	Countries reporting the cost of laboratory supplies, reagents and consumables as high-cost drivers	11 (85%)
	Cost of supply chain and procurement	Countries reporting the cost of supply chain and procurement as high-cost drivers	8 (62%)
	Cost of maintenance contract	Countries reporting the cost of equipment maintenance contracts as high-cost drivers	8 (62%)
Procurement	Forecasting	Forecasting conducted by the sequencing laboratory	Illumina=8 (62%) ONT=10 (77%) ThermoFisher=4 (31%) MGI/BGI=3 (23%)
	Procurement	Procurement conducted by the sequencing laboratory	Illumina=8 (62%) ONT=8 (62%) ThermoFisher=3 (23%) MGI/BGI=2 (15%)
	Purchasing of sequencing machine	Countries purchasing sequencing machine and related equipment from in-country distributor for at least one platform	9 (69%)
	Purchasing of reagents	Countries purchasing reagents from an in-country distributor for at least one platform	9 (69%)
	Purchasing of consumables	Countries purchasing consumables from an in-country distributor for at least one platform	10 (77%)
	Re-supply time	Median of re-supply time between order and receipt for reagents and consumables (weeks)*	8.0 (IQR 6.2–9.0)
	Equipment lead time	Countries reporting equipment purchasing lead time as a high barrier to sequencing	9 (69%)
	Reagents and consumables purchasing lead time	Countries reporting reagents and consumables purchasing lead time as a high barrier to sequencing	10 (77%)
Stock availability	Reagents and consumables stock out	Countries reporting stock outputs of reagents and consumables in the past 6 months	3 (23%)
	Reagents and consumables stock availability	Countries reporting reagents and consumables stock availability as a high barrier to sequencing	8 (62%)
Storage, distribution and transport	Reagents expiry date on arrival	Countries reporting expiry date on arrival for reagents as a high barrier to sequencing	4 (31%)
	Cold chain maintenance	Countries reporting cold chain maintenance as a high barrier to sequencing	1 (8%)
	Distributor responsiveness	Countries reporting distributor responsiveness as a high barrier to sequencing	1 (8%)
	Customs clearance	Countries reporting customs clearance as a high barrier to sequencing	4 (31%)
Post-sales	Equipment breakdown	Countries experiencing equipment breakdown in the past 6 months	7 (54%)
	Equipment repair lead time	Countries reporting equipment repair lead time as a high barrier to sequencing	9 (69%)

* Median (IQR).

ONT, Oxford Nanopore Technologies.

### Qualitative interviews with manufacturers

We adhered to the Consolidated criteria for Reporting Qualitative research checklist to report our qualitative methodology and findings^15^ (online supplemental appendix 2). We conducted 1-hour semi-structured interviews with the three major NGS manufacturers supplying 78% of the sequencing capacity in countries assessed to examine challenges, barriers and solutions related to PSM of equipment and consumables. These three manufacturers were selected as they supply the majority of the NGS-related equipment in the region. Manufacturers were engaged through written input, remote calls and face-to-face interviews. Participation was voluntary. Manufacturers requested access to the interview questions in advance to identify the appropriate respondents. The interview guide (online supplemental appendix 3) focused on the logistics and distribution side of PSM of genomic sequencing-related equipment. Interviews were conducted by two researchers. Two interviews occurred at the manufacturers’ offices. Interviews were audio-recorded using the Otter.ai app and field notes were taken. Oral informed consent was collected. One manufacturer preferred to respond to the questions in written form.

### Data analysis

Quantitative and qualitative data were mapped using the adapted Procurement and Supply Chain (PSC) framework developed by IQVIA Public Health.^16^ Quantitative assessment data from consumers were collected, reviewed and merged across institutions to generate national-level indicators. Data were analysed and visualised using Excel and Tableau Software (V.2023.1).^14^ Proportions are expressed as values and percentages. Likert data are shown as proportions of countries scoring above an indicated threshold (eg, a score of 4–5 on the Likert scale), with higher scores reflecting greater challenges. Continuous variables are displayed as mean or median and range or IQR.

Consistency of qualitative interviews was checked by the two researchers. Written responses were reviewed and integrated into the pool of interviews, alongside additional data from grey literature. Triangulated data were analysed using thematic content analysis. A deductive approach was followed to group data into PSC framework categories. Codes and categories were compared between the two researchers and discussed to reach an agreement. Microsoft Word was used for deductive data coding and categorisation.

## Results

A total of 42 institutions conducting genomic sequencing, and 3 genomic sequencing manufacturers contributed data to this assessment. The consumer responses are presented as aggregated national-level data.

**Figure 1:**
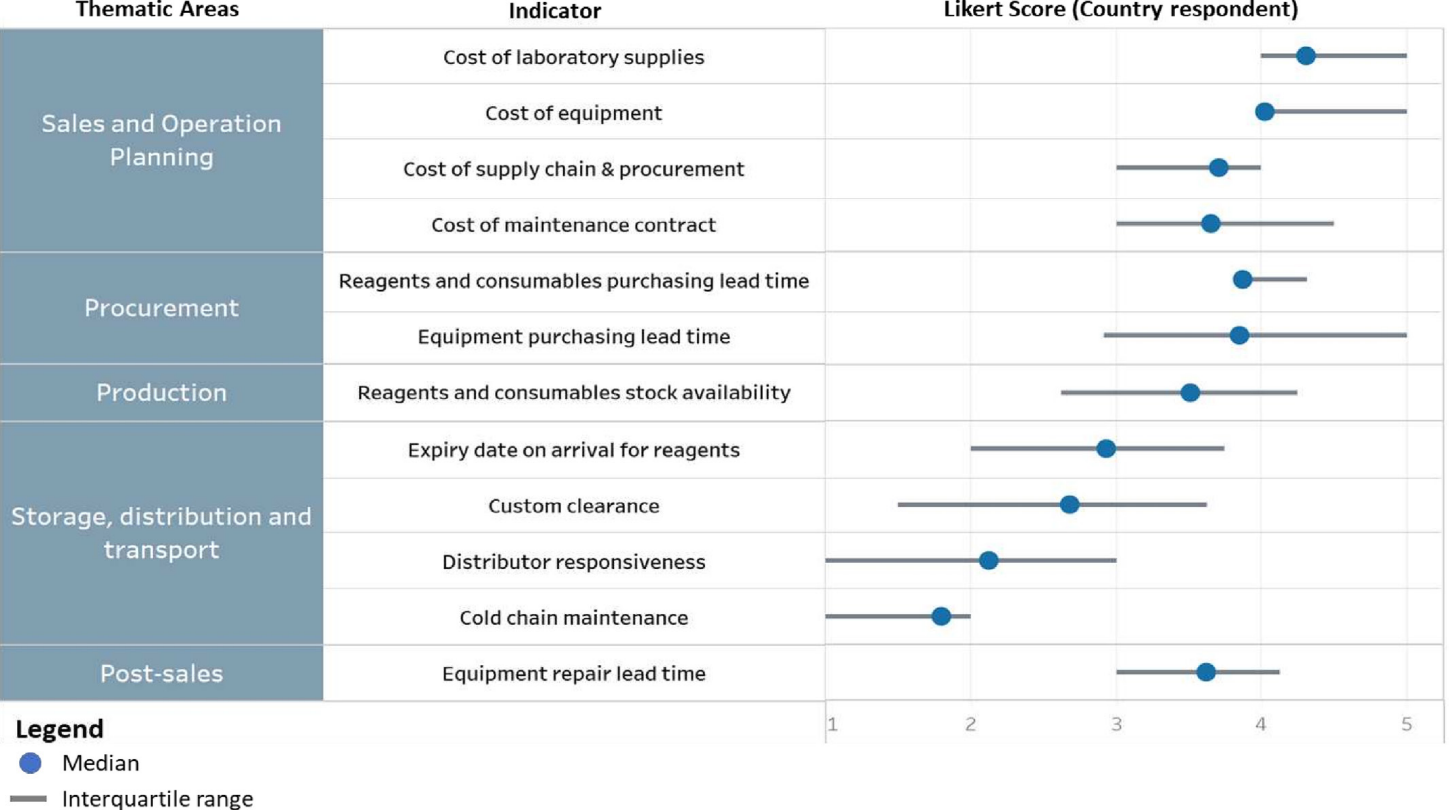
Major barriers to supply chain management of pathogen sequencing tools reported by 13 consumer countries.

Quantitative responses from pathogen genomics institutions (ie, consumers) and qualitative responses from manufacturers are profiled in tables1  2, and figure 1. Responses are profiled against PSC framework categories.

**Table 2:** Qualitative themes and illustrative quotes from three manufacturers

Thematic areas	Description	Quotes
Sales and Operation Planning	Explore the sales and operation planning strategies to facilitate access to genomic sequencing tools	**Interviewee 1**. In terms of barriers to entry, if a country couldn't afford [our product], I just think that [it is] what one of the many unique selling points, […] for the low-income countries. **Interviewee 2**. [We have] an access policy designed to promote access to genomic sequencing technology and data for research and clinical purposes, and to support scientific innovation and collaboration in the field of genomics. We are also currently enhancing the framework for equitable access for emerging markets, LMIC and global health agencies.
Procurement	Analyses the procurement mechanisms offered to customers	**Interviewee 1**. That [joint procurement], that will be the customers initiative. Yeah, [this] will not be from us, it will be from customer. How, how our researchers get together. Yeah, there is. Yeah, there is for them to initiate. […] So for us, we will treat every single order accordingly, same. I know that some of the customers [have], internally, to some collaboration among themselves […]. We have various research labs and somehow the researchers […] they have noticed they are using the same devices, so they actually they have been collaborating among themselves and then to place one single order to us. So, So, for us, we will treat every order the same. **Interviewee 3**. It all starts with [customers]. They will either send us [their] purchase order. So for purchase order we see that typically go through two rounds, either send it to the distributor or direct sales team itself.
Stock availability		This topic was not mentioned during the interviews
Storage, distribution and transport	Describes the different barriers and facilitators to deliver the goods to consumers	**Interviewee 1**. There are widely differing levels of taxation, which should make our product less attractive. **Interviewee 2**. [Supply chain challenges vary] across different countries. Some examples are questions and clarification on product specs, products, and import harmonized code. **Interviewee 3**. For the US and Europe, I would say even if you consider Africa, the amount of regulatory cases that you get, it is probably not going to be as diverse and as intense in terms of how to deal. Similarly, because there isn’t, I mean, at least for the Asia Pacific itself, there isn’t anything that is similar, that exists, say for example like the US and the European Union and the African Union. So for example, if you want to take like for Southeast Asia, like 12 or 13 countries, each one of the countries, they have their own rules and regulations, even though they have harmonized some of the rules, but basically it is still a work ongoing progress.
Post-sales	Explore customers services and technical support	**Interviewee 1**. So it’s only the license and warranty. Yeah, so the users will need to renew the license and warranty with us. Yeah, so with the license and warranty active, we will continue to support them from a technical perspective and from a customer service perspective. So if the device is down electronically, we will be able to replace a new device to them. Yeah. If the license and warranty is being renewed. **Interviewee 3**. Usually you’re trying to oversee the process, okay. […] Like the first line kind of support to a longer response. We will usually expect our distributors to at the very least try to understand and provide feedback to us.

### Sales and operation planning

**Figure 2:**
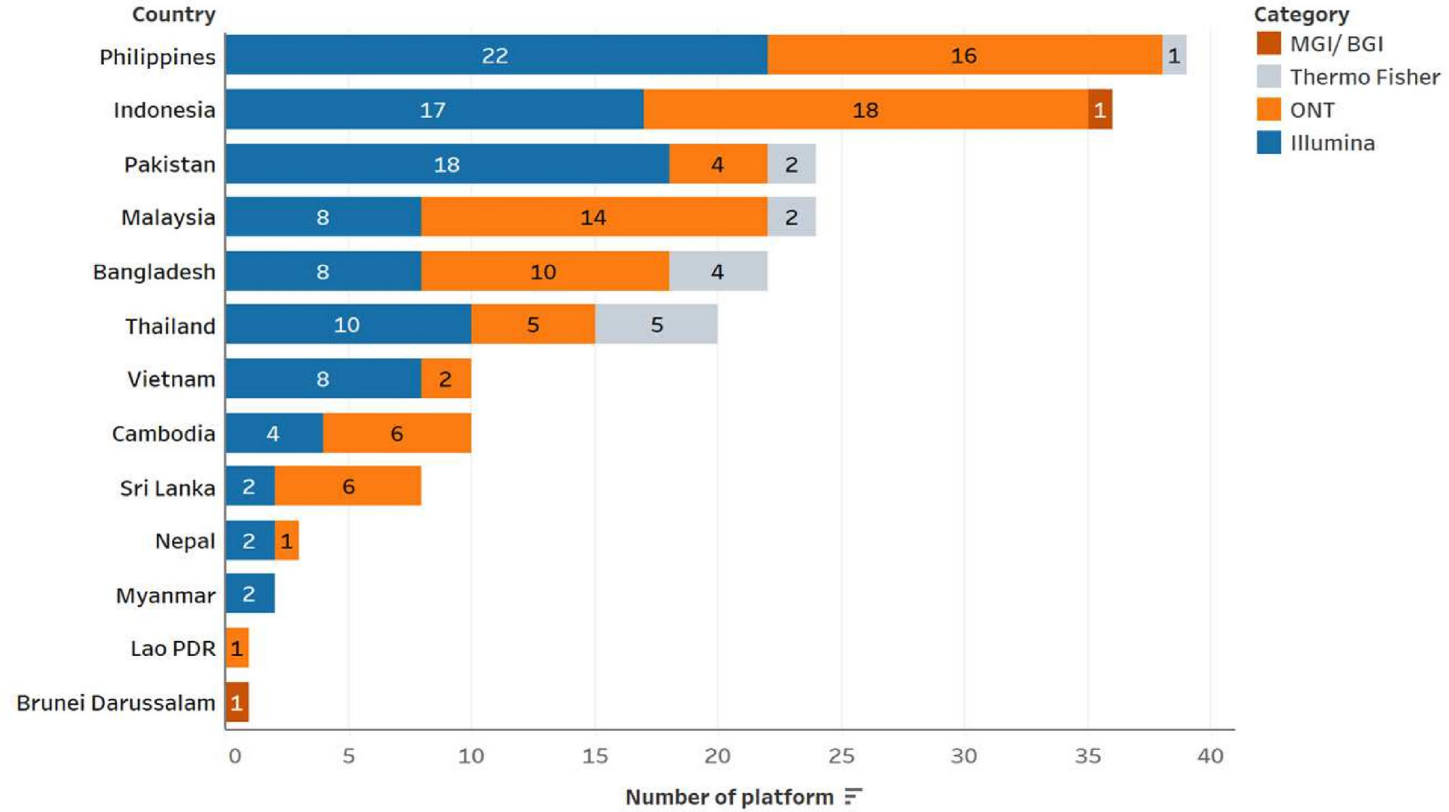
Number and type of sequencing platforms used by each consumer country for pathogen genomic surveillance. The Y-axis denotes the country, while the X-axis denotes the number of sequencing machines within each brand of platform. ONT, Oxford Nanopore Technologies.

All countries reported at least some national capacity for pathogen genomic surveillance.^14^ Just two manufacturers—Illumina (USA) and Oxford Nanopore Technologies (ONT) (UK) provide the majority of NGS platforms among countries assessed (figure 2). Another short-read technology manufacturer, MGI Tech (China), has a smaller market presence.

High costs of equipment and reagents were identified as a near-universal barrier to adoption and scale by countries (figure 1). Consumers perceived the cost of laboratory equipment, consumables and supplies as major cost barriers for pathogen genomic sequencing.

Nearly two-thirds of countries also identified maintenance contract costs as a barrier. In some countries, currency depreciation against the US dollar further drives up the costs.

Manufacturers have sales strategies to facilitate access to NGS tools in the region to address PSM issues. All three manufacturers expressed the intention of expanding their physical presence in the region to better support countries. Manufacturers support collaborations with academia, NGOs and private institutions to better tailor genomics applications for the region. All companies have established regional logistics hubs and/or satellite warehouses to strengthen their supply chains. Two of them are owned by the manufacturers and one is run by a third-party logistics provider. Interviewees also discussed ongoing innovations to facilitate access to pathogen sequencing in the region, such as leasing and discounted pricing schemes for low-resourced countries, which potentially reduce import taxes.

One such example of the discounted pricing scheme available is the Illumina Global Access Initiative^17^ to support access to NGS-related equipment for public health in low-resourced countries. This initiative offers combined discounts on different products from standard offerings in the price range of US$60–100 per sample. Similar pricing schemes are in development with other manufacturers.

### Procurement

Procurement and forecasting for pathogen sequencing equipment are largely conducted by individual facilities except for Indonesia (table 1). Most countries (72%) purchase equipment, reagents and non-consumable reagents for their platforms through in-country distributors. India, Nepal, Pakistan, the Philippines and Thailand also used the WHO as a group purchasing organisation. Brunei, which is only starting to conduct pathogen genomic surveillance, currently relies on external donations for its pathogen genomic sequencing capacity.

More than two-thirds of countries highlight concerns regarding long purchasing lead times for genomic sequencing-related equipment. The average re-supply time between the order and receipt for reagents and consumables is about 8 weeks (IQR 6.2–9.0) (table 1). Results indicate a wide range of re-supply times across the region, with Cambodia, Malaysia, Nepal, Pakistan, Thailand and Vietnam reporting a re-supply time from ordering to arrival in labs of less than 8 weeks, and Bangladesh, Brunei, Indonesia, Laos, the Philippines and Sri Lanka reporting a re-supply time of 8–12 weeks. At the institutional or laboratory level, geographical distances between laboratories and ports of entry within the same country also contribute to varying shipping costs. This further widens the price inequity and access to the technology.

Qualitative interviews with the manufacturers revealed that there are different procurement models. Based on the customer’s needs and profile, procurement of genomic sequencing equipment and reagents can be placed either directly through manufacturers or distributors. Occasionally, pooled procurement orders have been made by academic institutions, but not at a national level, based on aggregated forecasts. Importantly, most procurement takes place as small volume single-pathogen focused orders between individual laboratories and manufacturers, rather than through pooled national forecasts for multi-pathogen use cases, which drives costs higher.

### Stock availability

Stockouts represent an additional bottleneck for countries. Stock availability for reagents and consumables at the manufacturer level is viewed by consumers as an implementation barrier in over 60% of countries (table 1). The findings illustrate varying levels of concern among consumer countries regarding equipment and reagent stockouts. Bangladesh considered stockouts the least challenging, while Sri Lanka and Indonesia expressed that stockouts posed substantial challenges.

### Storage, distribution and transport

All three manufacturers have logistics hubs in South and Southeast Asia to facilitate the export of sequencing products. One manufacturer recently expanded its collaboration with a healthcare logistics company by storing their equipment in a Singapore-based distribution facility. This facility can deliver pathogen sequencing products within 24–48 hours across the region.

Manufacturer interviews highlighted the importance of building a widespread network of distributors to cater to the surrounding countries. Across 13 countries, there are 3 major genomic sequencing manufacturers with a network of 18 distributors (17–19) and growing. It was reported that although the number of distributors in each country is contingent on local regulations, existing distributors are of paramount importance to all manufacturers, serving as importers-of-record and offering local customer support services, including equipment installation and maintenance when required. Selection criteria for distributors are clearly defined by the manufacturers and encompass factors such as experience, familiarity with local customs procedures and regulations, market penetration, sales proficiency, logistics support and cost-effectiveness.

Manufacturers revealed that they provide training tailored to the distributor’s maturity and business domain. This training encompasses product knowledge, technical understanding, technology usage and workflow. Distributors continuously receive refresher training and ongoing support to equip them in providing technical assistance to their customers. Customers frequently reach out to distributors directly for support, often due to language and accessibility considerations. Notably, all manufacturers reported positive relationships with their distributors in this region, and distributor responsiveness is not perceived by labs as a significant barrier (92%).

Manufacturers raised concerns about the extended and fluctuating import procedures for genomic sequencing platforms, reagents and related supplies, encompassing the authorisation application. The complexity of this process varies according to market maturity, with all interviewees emphasising its impact on turnaround times. Notably, one manufacturer pointed out that the variability is more pronounced in the region compared with Europe.

Manufacturers reported wide variability in the interpretation and enforcement of regulatory guidelines and frameworks for importation across countries. Some countries categorise pathogen sequencing items as medical devices or in vitro diagnostics, which requires more stringent regulatory oversight and consequently leads to importation delay. All manufacturers have a regulatory affairs team to help them navigate the complexity of the regulatory processes.

Customs clearance presents a significant bottleneck for manufacturers. Although only 31% of countries considered it a significant barrier, many manufacturers commonly regard it as a critical obstacle. Manufacturers described a highly variable customs clearance process across the region, citing unclear guidelines from local authorities and inconsistent enforcement by customs officers. Customs clearance is overseen by importers-of-record, responsible for ensuring compliance with the requirements at the port of entry. Importers of record can be either distributors or customers in the absence of distributors. Some manufacturers act as importers-of-record, where they have warehouses, with one manufacturer mentioning collaboration with a customs broker for insights into country regulations.

Duties and taxes play a significant role in the increased cost of NGS goods. Manufacturers raised concerns about varying taxation mechanisms across countries, which can increase costs, especially for resource-constrained countries. In certain countries, high customs duties can double the price of smaller sequencing machines. Complicated tax exemption processes also delay the importation.

While reagents are temperature-sensitive, survey respondents did not report any cold chain breaches, and interviewees did not express concerns about maintaining cold chain integrity during delivery and transport.

### Post-sales support

Manufacturers’ interviews described that they maintain customer service support post-sales, and technical support teams are available to address and resolve customer inquiries when distributors are unable to do so. These inquiries encompass product specifications, pricing, shipment localisation and technical queries related to workflows and reagents. For certain platforms, distributors may also quote equipment maintenance.

Equipment breakdowns are common across all sequencing platforms. Over half (7/13) of countries experienced breakdowns within the preceding 6 months of filling the survey, with over two-thirds (9/13) of countries perceiving repair lead times as a high barrier for pathogen sequencing. Manufacturers reported that service contracts for sequencing platforms can be established either directly with the manufacturer or distributors, depending on the platform. In the event of unresolved issues, escalation to the manufacturers is possible for all platforms. Moreover, a typical warranty period of 1 year can be negotiated or renewed for an extended duration.

## Discussion

We assessed PSM challenges related to pathogen genomic surveillance among 13 countries in South and Southeast Asia with varying genomic sequencing capacity. Across all countries, national capacity exists to conduct genomic sequencing, with much of this built during the COVID-19 pandemic.^14^ Despite the importance of genomics in enhancing early pathogen detection and response,^5^ our assessment suggests PSM challenges remain a major barrier limiting the pace of pathogen genomics implementation.

Our findings note that the market for pathogen genomic surveillance in South and Southeast Asia is dominated by just three manufacturers, with two manufacturers (Illumina and ONT) supporting the bulk of NGS platforms. Costs associated with the procurement of equipment and reagents/consumables were identified as a near-universal barrier to adoption and scale. There is currently limited price transparency and visibility on what networks of country distributors charge to end-users. This is compounded by sole distributor licences for sequencing platforms, which limit competition. Given that low- and middle-income countries are paying substantially greater cost-per-sequence than wealthy countries,^11^ more detailed information on how price points change as the product moves from manufacturers to laboratories is essential. Higher pricing transparency will enable greater negotiation power of end-users, with the potential to drive down costs. The WHO Genomics Costing Tool, primarily designed to support countries in budgeting for genomic surveillance, can also be used as a price comparison tool and encourage pricing transparency across laboratories, countries and the region.^18^ In over two-thirds of countries, forecasting and procurement take place between individual laboratories and manufacturers or distributors, with national and regional forecasting largely absent. While distributors do sometimes conduct pooled procurement, this often extends waiting times, with some laboratories receiving supplies much later than when orders were submitted. Notably, pathogen sequencing equipment and reagents have recently been listed on global supply catalogues, which creates opportunities for price reductions through aggregating demand and pooling procurement across countries.^19 20^ The presence of the WHO providing procurement support is critical in maintaining accessibility to reagents and consumables. Collaborations between public health institutions conducting pathogen genomics and multilateral or non-governmental organisations that have tax exemption on NGS-related equipment are also a possible channel to reduce costs and improve access. However, maintenance contract costs pose a significant sustainability challenge in two-thirds of the countries. This may be attributed to several factors, including limited domestic budget allocations beyond the initial procurement period and the absence of dedicated funding mechanisms following donor-supported procurement or donations.

At the laboratory level, end users are also actively exploring other cost reduction strategies such as volume miniaturisation. Sequencing using miniaturised volumes has shown comparable results obtained using manufacturer-recommended full volumes.^21 22^ Further studies are needed to validate this strategy for other pathogens, sequencing platforms and reagents, but it could potentially be a cost-effective method for sequencing, especially in lower-resourced settings.

Distribution systems among countries assessed remain inadequate, with purchasing and re-supply lead times averaging 2 months and stock availability identified as a concern in most countries. Despite most platforms being recently introduced, more than half of the countries had experienced equipment breakdowns in the 6 months before the assessment, with over two-thirds of countries noting lengthy time-to-repair times as a concern. Improvements in logistics and distribution systems for sequencing products would not only improve pathogen genomic surveillance but also have a positive effect on emerging human genomics applications.

Heterogeneous import procedures and regulations were identified by manufacturers as contributing to high costs and delivery delays. Customs clearance delays both increase costs and affect the ability to use time-sensitive biological reagents efficiently. Furthermore, taxes and duties vary across countries, adding to the total costs and making it less attractive for countries to invest in NGS-related equipment. Novel procurement mechanisms such as leasing contracts are currently being explored by manufacturers as a strategy to enable tax exemption. Streamlining and strengthening existing regulatory processes within the countries is essential to improve access to NGS-related equipment. Current trade agreements and harmonisation efforts within regional associations like the Association of Southeast Asian Nations (ASEAN) require further evaluation regarding their impact on pandemic preparedness and genomic sequencing purchases.^23^ Finally, previous studies have shown that countries face additional clearance and distribution challenges related to physical infrastructure, gaps in effective distribution networks and political and environmental barriers.^24^

Efforts are currently underway in the region to mitigate these PSM challenges. Manufacturers are employing regional manufacturing, warehousing and distribution strategies to enhance resilience. Mechanisms to improve high-quality after-sales service support are crucial in addressing equipment service breakdown, maintenance issues and minimising downtime. Appointed distributors have selection criteria to support manufacturers, especially importers of records who are familiar with local regulations and for technical support. Manufacturers have also implemented improvements in post-sales services by setting up dedicated customer service and technical support teams. Digital technologies have the potential to improve the efficiency of the supply chain by enhancing visibility of the supply chain network, facilitating collaboration between stakeholders to conduct joint forecasting, producing more granular data for accurate decision-making, improving procurement processes through electronic systems and also using artificial learning (AI) to improve demand forecasts.^25^

Regionally, there are significant opportunities to strengthen coordination and engage the diverse stakeholders involved in the procurement and supply chain management of genomic-sequencing related equipment. WHO regional offices or ASEAN could host a multisectoral platform that convenes technical experts, manufacturers, distributors, funders, academia and policymakers to jointly address these challenges and co-develop contextually appropriate solutions for the Asian region.

Through such a platform, countries can jointly address cross-border barriers, including protracted customs procedures and unharmonised regulatory requirements that impede timely access to genomic technologies. It provides a structured forum for manufacturers, distributors and national authorities to explore pooled or bundled procurement arrangements, which can lower costs and foster stronger public–private partnerships through economies of scale. Laboratory end users can also contribute critical insights on operational challenges, such as difficulties in volume forecasting, and help co-develop more streamlined procurement pathways and performance-tracking systems.

This approach has precedent in the African region, where the Science for Africa Foundation convened a multisectoral, multistakeholder workshop and subsequently developed a 5-year implementation plan to address challenges in PSM and improve access to genomic technologies.^26^

Our paper focuses on South and Southeast Asia, but it is important to note that similar issues occur in other regions in the world. Long purchasing lead times, complicated customs clearance, inefficient distribution, high cost of equipment, lack of price transparency and unstable funding, which is reliant on external support, are some of the challenges faced in the African region.^27^ As PSM challenges are global in nature, this underscores the critical need to address and resolve PSM bottlenecks to enhance access to NGS technology. Although the study identified several practical solutions that could be implemented by end users and manufacturers, resolving broader systemic challenges will require greater governmental oversight and political commitment.

The market landscape for pathogen genomics is expanding rapidly, with new market entrants^28^ and its potential scope for clinical application^29^ potentially influencing prices. Further studies are needed to analyse costs, strategic planning, procurement, human resource capacity and distribution networks. This includes organisational structures, extending beyond platform manufacturers’ typical boundaries to include various third-party reagents playing a role in the sequencing process. Anticipating increased demand is critical as pathogen genomic sequencing shifts towards becoming a clinical diagnostic tool^30^ and increasingly being advocated to be integrated in wider existing surveillance systems.^31^

While this study comprehensively assesses PSM challenges among the major manufacturers and institutions contributing to pathogen genomics surveillance in the region, there are several limitations. First, the study predominantly engaged pathogen sequencing country labs (ie, consumers) and sequencing platform manufacturers, lacking involvement from policymakers, sequencing platform distributors, raw material suppliers, third-party reagent manufacturers and distributors, or logistics companies. Second, the absence of in-depth country-specific data limits a comprehensive understanding of how cost, forecasting and procurement mechanisms affect supply chain management. Third, the study provides a snapshot of the situation within the PSM domain during and immediately after the COVID-19 crisis. Continuous tracking of PSM indicators is essential for comprehending the long-term effects and changes within the supply chain. This study acknowledges that other factors can contribute to access to NGS technology, such as bioinformatics and the related IT infrastructure, among others. These factors can be further assessed in future studies.

## Conclusion

In summary, pathogen genomic surveillance using NGS is a ground-breaking new technology, with the ability to overcome current inequalities in early infectious disease detection, monitoring and response capacity. To realise this potential, overcoming PSM barriers among high disease-burden low-resource settings in South and Southeast Asia is essential. A set of recommendations to enhance resiliency and streamline the NGS supply chain has been highlighted to address pressing concerns (table 3). Coordinated efforts to enhance the efficiency of PSM systems, alongside improving laboratory capacity, quality assurance, bioinformatics and cross-country data sharing, are essential to improving pathogen genomic surveillance capacity in low-resource settings.

**Table 3:** Recommendations to strengthen the supply chain for pathogen genomic sequencing in Asia

Thematic areas	Key constraints	Key recommendations
Sales and operation planning	Cost to access genomics sequencing for low- and middle-income countries	Ensure transparent and affordable pricing for genomic commodities from suppliers, by: Developing and maintaining an online pricing database for genomics and molecular diagnostics Conducting cost comparison, evaluating genomics alongside molecular dx for specific use-cases or pathogen groupings Investigate and address price disparities across South and Southeast Asia Map end-to-end sequencing costs in select countries to identify cost drivers that should be targeted for reduction in select countries Advocate for investment to implement specific access pricing strategies in low- and middle-income countries
Procurement	Long purchasing lead times for equipment, reagents and non-consumables	Engage in further discussions with country partners to thoroughly understand their forecasting and procurement needs Develop new forecasting tools to enhance accuracy and efficiency Implement a performance tracking system Maintain an ongoing dialogue with manufacturers to reduce purchasing lead times Explore innovative approaches for identifying and deliberating on alternative procurement methods, including but not limited to pooled procurement, leasing arrangements and batch procurement models Explore best practices regarding alternative procurement modalities such as platform leasing and implications on supply chain barriers (importation fees, taxes etc)
Production	Stock availability	Implement new technology to monitor the production of sequencing platforms, reagents and consumables in real-time Engagement of manufacturers of sequencing products and more general lab commodities/products that are essential to completing a sequence and keeping a lab well-stocked
Storage, distribution and transport	Customs and tax challenges due to heterogeneity of import regulation and guidelines	Establish a regional working group to bolster end-to-end supply chains, minimising disruptions related to supplies for sequencing efforts in the region Support the development of national investment cases, guidelines and policies Facilitate genomic system design and demand forecasts Streamline and accelerate procurement processes (regulatory requirements, customs clearance, permits, tax waivers) Implement a system to monitor and evaluate delivery performance Facilitate product donations from genomics suppliers
Post-sales	Equipment breakdown and maintenance	Strengthen post-sales teams to improve customer support Ensure that service level agreements encompass maintenance and technical support provisions

## Supplementary material

10.1136/bmjgh-2025-019241 online supplemental appendix 1

10.1136/bmjgh-2025-019241 online supplemental appendix 2

10.1136/bmjgh-2025-019241 online supplemental appendix 3

10.1136/bmjgh-2025-019241 online supplemental file 1

## Data Availability

Data are available upon reasonable request.
