# Supplementary material for: Strengthening supply chains for pathogen genomic surveillance in Asia
Source: BMJ Glob Health. 2026 Feb 6;11(2):e019241. doi: 10.1136/bmjgh-2025-019241 (PMC12887454; doi:10.1136/bmjgh-2025-019241)
Supplement: online supplemental appendix 1 [file bmjgh-11-2-s001.docx]

### Appendix1- List of participating countries and institutions

| **Country income level**  **(World Bank classification)** | **Country** | **Institution** |
| --- | --- | --- |
| Lower-middle Income | Bangladesh | 1. Child Health Research Foundation (CHRF) |
|  |  | 2. Institute of Epidemiology, Disease Control and Research (IEDCR, Bangladesh) |
|  |  | 3. International Centre for Diarrhoeal Disease Research (icddr,b) |
| High Income | Brunei | 4. Department of Laboratory Services, Ministry of Health |
| Lower-middle Income | Cambodia | 5. Institute Pasteur Cambodia (IPC) |
|  |  | 6. International Center of Excellence in Research (ICER), National Institutes of Health |
|  |  | 7. National Institute of Public Health (NIPH) |
| Upper-middle Income | Indonesia | 8. Health Development Policy Agency, Ministry of Health |
|  |  | 9. Biomedical and Genome Science Initiative (BGSI), Ministry of Health |
| Lower-middle Income | Lao PDR | 10. Lao-Oxford University-Mahosot Hospital-Wellcome Trust Research Unit (LOMWRU) |
|  |  | 11. National Centre for Laboratory and Epidemiology (NCLE) |
| Lower-middle Income | Myanmar | 12. Department of Medical Research, Ministry of Health |
|  |  | 13. National Health Laboratory (NHL) Department of Medical Service, Ministry of Health |
| Upper-middle Income | Malaysia | 14. Malaysia Genome and Vaccine Institute (MGVI), National Institutes of Biotechnology Malaysia (NIBM) |
|  |  | 15. Institute for Medical Research (IMR), Ministry of Health Malaysia |
|  |  | 16. Hospital Canselor Tuanku Muhriz UKM (HCTM) |
|  |  | 17. Universiti Kebangsaan Malaysia (UKM) |
|  |  | 18. Universiti Malaya (UM) |
|  |  | 19. Tropical Infectious Diseases Research and Education Centre (TIDREC), University Malaya |
|  |  | 20. Universiti Teknologi MARA (UiTM) |
|  |  | 21. Hospital Sultan Abdul Aziz Shah (HSAAS) |
|  |  | 22. Universiti Putra Malaysia (UPM) |
|  |  | 23. Universiti Sains Malaysia (USM) |
|  |  | 24. International Islamic University Malaysia (IIUM) |
|  |  | 25. Universiti Malaysia Sarawak (UNIMAS) |
|  |  | 26. Universiti Malaysia Sabah (UMS) |
|  |  | 27. National Public Health Laboratory |
| Lower-middle Income | Nepal | 28. WHO (Nepal Country Office) |
|  |  | 29. National Public Health Laboratory |
| Lower-middle Income | Pakistan | 30. National Institute of Health (NIH) |
|  |  | 31. Aga Khan University (AKU, Pakistan) |
| Lower-middle Income | Philippines | 32. Research Institute for Tropical Medicine (RITM) |
|  |  | 33. Philippine Genome Center (PGC), University of the Philippines |
| Lower-middle Income | Sri Lanka | 34. Ministry of Health |
|  |  | 35. University of Sri Jayewardenepura |
| Upper-middle Income | Thailand | 36. Department of Medical Sciences, Ministry of Health |
|  |  | 37. Mahidol University |
|  |  | 38. COVID-19 Network Investigations Alliance |
| Lower-middle Income | Vietnam | 39. National Institute of Hygiene and Epidemiology |
|  |  | 40. Oxford University of Clinical Research Unit |
|  |  | 41. Institute Pasteur, Ho Chi Minh City (IP HCMC) |
|  |  | 42. Institute Pasteur, Nha Trang (IP Nha Trang) |
