# Supplementary material for: Strengthening supply chains for pathogen genomic surveillance in Asia
Source: BMJ Glob Health. 2026 Feb 6;11(2):e019241. doi: 10.1136/bmjgh-2025-019241 (PMC12887454; doi:10.1136/bmjgh-2025-019241)
Supplement: online supplemental appendix 3 [file bmjgh-11-2-s003.docx]

**Appendix 3 – Interview guide with manufacturers**

**APGI Supply Chain & Procurement Workstream**

Thank you so much for participating in this interview. The purpose of this interview is to better understand the supply chain dynamics for genomic sequencing in the region, explore the challenges and barriers that you are facing and discuss sustainable solutions.

Your feedback will allow us to get an in-depth understanding of the current state of the supply chain for genomic sequencing from a manufacturer’s perspective. It will also help document planned solutions and areas where further work may be required.

Please note that the information gathered is intended to be shared and published in an aggregated and anonymized manner.

| **Theme** | **Question number** | **Interview guide/questions** | **Prompt** |
| --- | --- | --- | --- |
| **Generic** |  | Can you provide a short summary of the procurement and supply chain for NGS equipment and reagents? | Can you explain how customer order the products, how do you receive the order, how do you manage inventory, delivery and specific customers’ requests before and post-sale? |
|  |  | Does [your] supply chain processes differ between your different types of genomics products (i.e pathogen genomic sequencing, human genomic sequencing…)> | If yes, can you explain those differences |
|  |  | What are the types of clients who access [your] NGS products? | For example, Publics Labs, private labs, NGOs, International Organizations, universities…? |
|  |  | Would you be able to share country-level information on what **type** and **how many** of sequencing equipment are currently being utilized in the various Asian countries? | What are the most common pathogen sequencing reagents utilized in the South and South East Asian region? |
|  |  | What are the main challenges and barriers [your] face in the genomics sequencing supply chain? |  |
|  |  | What are the main applications your NGS products are being used for - e.g. research, surveillance, diagnostics? Are there any import taxes or fees for different types of intended use? | Are there any plans to expand to diagnostics?  Is there any specific reason as to why you would like to shift to diagnostics? Can you explain more about the process and future plans?  Are there any concerns on the regulatory procedures? |
|  |  | Are your products subject to review and approval by national regulatory authorities? What are the regulatory requirements? | Would [your] be interested to know more on the regulatory procedures to get NGS equipment classified as diagnostics? |
|  |  | Do the challenges differ between Asia and other regions of the world? If yes, could you explain. | Do they differ within Asia? If yes, could you explain. |
| **Distributor relationship** |  | What is the role for the in-country distributor or channel partner? | What are the responsibilities of the in-country distributor or channel partner? |
|  |  | How do you perceive your relationships with the distributors in the region? |  |
|  |  | What determines the manufacturer’s choice to engage with distributors? | Do you have any criteria by which you determine your distributors? |
|  |  | Do you have control/input over and visibility of the prices distributors charge to customers? | What are the mechanisms to control the prices charge to customers?  Is there any guidance document that you provide to your distributors with regards to price control and other inputs? |
|  |  | In what ways, if at all, does the NGS market require more investment on resources/training for the distributors? |  |
| **Custom clearance** |  | Who handles the customs clearance? | Customer, distributor, shipping company? |
|  |  | Do you encounter any issues regarding customs clearance? |  |
| **Transportation and shipping** |  | Which shipping company do you work with? | How is the relationship with the shipping company? |
|  |  | Do you face challenges regarding cold chain maintenance and arrival expiry dates? | What can manufacturers do to help countries facing cold chain maintenance and arrival expiry dates due to delivery issues? |
| **Customers services** |  | Do customers approach you or the distributor for troubleshooting? |  |
|  |  | What are the most frequent customer requests that you receive- **pre-sale**? |  |
|  |  | What are the most frequent customer requests that you receive – **post sale**? |  |
|  |  | Do you have a feedback mechanism for distributors and consumers to send back queries, performance/quality issues on products? | How can the feedback process to send back queries, performance/quality issues on products be improved? Who is responsible? |
| **Equipment maintenance** |  | Is the Equipment maintenance provided by you and/or the distributor? | Is the Equipment maintenance contract built into the purchasing order? |
| **Cost** |  | How do you define the price? | What are the major cost drivers?  What are the criteria/consideration of the price point? For eg volume, location, public vs private buyer |
|  |  | Are there any modifications to your approach or price structure for low-and-middle income countries? | Do you offer discounted prices for low-and-middle income countries?  Are there any plans for a tiered pricing system, similar to pharmaceutical companies, for LMICs countries for genomics sequencing-related equipment? |
| **Joint procurement** |  | How do you approach requests for joint procurement in the same country? | Do you receive requests for joint procurement?  If yes, are they all evaluated? Are they all feasible?  Do you have internal procedures with regards to joint procurement? |
| **APGI** |  | How do you think our initiative can help with the supply chain challenges in the region? | What are some key gaps and challenges you face in knowledge/network on these new geographies and/or applications you are serving? |
|  |  | Apart us, do you have existing engagement with partners to identify market interventions to improve access in Asia? |  |
|  |  | Would you be willing to participate in a dedicated working group with other stakeholders – labs, distributors, policymakers, health administrators… to work on supply chain and procurement challenges? |  |
|  |  | Would you agree that we contact distributors? | Could you give us the contact details of the distributors? |
|  |  | Would you agree to participate in follow-up interviews or surveys about supply chain and procurement? |  |

Thank you so much for participating in this interview again. We are most grateful for your sharing and let me once again assure you that this interview is completely confidential. We will remove anything that you do not want to share. Before we end this session, do you have anything else want to share with us?
