# Supplementary material for: Strengthening supply chains for pathogen genomic surveillance in Asia
Source: BMJ Glob Health. 2026 Feb 6;11(2):e019241. doi: 10.1136/bmjgh-2025-019241 (PMC12887454; doi:10.1136/bmjgh-2025-019241)
Supplement: online supplemental file 1 [file bmjgh-11-2-s004.docx]

### BMJ Global Health Author Reflexivity Statement

Adapted from Morton, B., Vercueil, A., Masekela, R., Heinz, E., Reimer, L., Saleh, S., Kalinga, C., Seekles, M., Biccard, B., Chakaya, J., Abimbola, S., Obasi, A. and Oriyo, N. (2022), Consensus statement on measures to promote equitable authorship in the publication of research from international partnerships. Anaesthesia, 77: 264-276. <https://doi.org/10.1111/anae.15597>

| **Study conceptualisation** | |
| --- | --- |
| 1. How does this study address local research and policy priorities? | The study aligns with local research and policy priorities by engaging local stakeholders through the survey, and is further informed by an earlier landscaping assessment that outlined key local findings. |
| 1. How were local researchers involved in study design? | Local researchers were involved in the data collection, analysing and validation of the study and writing of the manuscript. |
| **Research management** | |
| 1. How has funding been used to support the local research team(s)? | Funding from project was used to conduct research and training activities participated by local researchers, or nominations from local researchers. |
| **Data acquisition and analysis** | |
| 1. How are research staff who conducted data collection acknowledged? | Research staff which conducted data collection is acknowledged as co-authors in the study. |
| 1. How have members of the research partnership been provided with access to study data? | Study data was shared through encrypted files and secure shared folders. |
| 1. How were data used to develop analytical skills within the partnership? | Study data was used to jointly analyse and validate the findings and refine analytical frameworks. This is a collaborative process and resulted in strengthened partnership. |
| **Data interpretation** | |
| 1. How have research partners collaborated in interpreting study data? | Research partners collaborated by jointly reviewing datasets, discussing emerging patterns, and reaching consensus on the interpretation of key findings through regular analysis meetings. |
| **Drafting and revising for intellectual content** | |
| 1. How were research partners supported to develop writing skills? | Writing skills were strengthened through collaborative drafting of study outputs, where partners co-wrote or edited sections. |
| 1. How will research products be shared to address local needs? | Research products will be disseminated through tailored briefs, stakeholder presentations, and open-access reports to ensure findings directly address and inform local needs. |
| **Authorship** | |
| 1. How is the leadership, contribution and ownership of this work by LMIC researchers recognised within the authorship? | Authorship decisions were made transparently and collaboratively, ensuring that LMIC researchers’ substantive contributions to the conception, analysis, and writing of the study were appropriately recognised. |
| 1. How have early career researchers across the partnership been included within the authorship team? | The partnership intentionally included early career researchers in the authorship team by providing opportunities to lead sections of the analysis, draft manuscript components, and engage in the revision process. |
| 1. How has gender balance been addressed within the authorship? | Gender balance was addressed through transparent authorship criteria and inclusive collaboration practices that enabled equitable contribution and recognition across genders. |
| **Training** | |
| 1. How has the project contributed to training of LMIC researchers? | The project contributed to training LMIC researchers through hands-on involvement in study design, data collection, analysis, and dissemination, supported by mentorship from senior partners. |
| **Infrastructure** | |
| 1. How has the project contributed to improvements in local infrastructure? | The project contributed to improvements in local infrastructure by providing actionable recommendations to improve access to genomic technologies. |
| **Governance** | |
| 1. What safeguarding procedures were used to protect local study participants and researchers? | Safeguarding procedures combined participant protection and researcher safety: informed consent, privacy measures, secure data management, ethical approvals, and regular monitoring to address risks during the study. |
